# Macrophage Phenotype in Combination with Tumor Microbiome Composition Predicts RCC Patients’ Survival: A Pilot Study

**DOI:** 10.3390/biomedicines10071516

**Published:** 2022-06-27

**Authors:** Olga V. Kovaleva, Polina Podlesnaya, Maxim Sorokin, Valeria Mochalnikova, Vladimir Kataev, Yuriy A. Khlopko, Andrey O. Plotnikov, Ivan S. Stilidi, Nikolay E. Kushlinskii, Alexei Gratchev

**Affiliations:** 1 N.N. Blokhin National Medical Research Center of Oncology, 115478 Moscow, Russia; ovkovaleva@gmail.com (O.V.K.); polina.pod@yandex.ru (P.P.); maxsorokin9@gmail.com (M.S.); mochalnikova70@yandex.ru (V.M.); director@ronc.ru (I.S.S.); kne3108@gmail.com (N.E.K.); 2 Institute for Cellular and Intracellular Symbiosis of the Ural Branch of the Russian Academy of Sciences, 460000 Orenburg, Russia; vladimir0334@yandex.ru (V.K.); 140374@mail.ru (Y.A.K.); protoz@mail.ru (A.O.P.)

**Keywords:** renal cell carcinoma, prognosis, PU.1, microbiome, macrophage, stroma

## Abstract

The identification of new prognostic markers of renal cell carcinoma (RCC) is an urgent problem in oncourology. To investigate the potential prognostic significance of tumor microbiome and stromal inflammatory markers, we studied a cohort of 66 patients with RCC (23 clear cell RCC, 19 papillary RCC and 24 chromophobe RCC). The microbiome was analyzed in tumor and normal tissue by 16S rRNA amplicon sequencing. Characterization of the tumor stroma was performed using immunohistochemistry. A significant difference in alpha diversity was demonstrated between normal kidney tissue and all types of RCC. Further, we demonstrated that the bacterial burden was higher in adjacent normal tissue than in a tumor. For the first time, we demonstrated a significant correlation between bacterial burden and the content of PU.1+ macrophages and CD66b+ neutrophils in kidney tumors. Tumors with high content of PU.1+ cells and CD66b+ cells in the stroma were characterized by a lower bacterial burden. In the tumors with high bacterial burden, the number of PU.1+ cells and CD66b+ was associated with a poor prognosis. The identified associations indicate the great prognostic potential of a combined tumor microbiome and stromal cell analysis.

## 1. Introduction

Renal cell carcinoma (RCC) currently accounts for around 2–4% of all diagnosed tumors, with a trend towards an increase in the number of newly diagnosed cases [1,2,3,4]. The modern classification of RCC is based on morphological, genetic and molecular features and distinguishes 13 types, of which the most common are clear cell carcinoma (60–85%), papillary carcinoma (7–14%), chromophobe cancer (4–10%), oncocytoma (2–5%) and collecting duct cancer (1–2%) [5]. Chromophobe RCC differs from other histotypes due to its better prognosis. Tumors of the papillary type often occur with bilateral lesions, are characterized by the presence of multiple lesions and are associated with acquired chronic kidney disease [6]. Papillary cancer, in contrast to clear cell carcinoma, responds less well to systemic targeted and immunotherapy (VEGF, mTOR, TIK inhibitors). If, for clear cell RCC, the response is up to 85% of cases, then for papillary RCC, it is no more than 15% [7]. Thus far, the treatment of RCC is selected according to a risk assessment that considers clinical criteria and includes the abovementioned targeted and immunotherapy drugs [8]. This approach, however, is not personalized and does not take into account the molecular peculiarities of the tumor. Molecular markers that are being explored for their prognostic value in RCC include *VHL*, *PBRM1*, *BAP1* and *SETD2*; however, none of these are validated for clinical use so far [9].

One of the features of malignant kidney tumors is their high immunogenicity. Advances achieved in the treatment of lung cancer and melanoma with immunotherapy suggest its positive effect also in the case of RCC. Indeed, in the case of metastatic clear cell RCC, immunotherapy has improved patient survival rates. Immunotherapy with check-point inhibitors can also be used in combination with tyrosine kinase inhibitors, which leads to a consistent benefit in RCC patients [10,11,12]. However, for many patients with RCC, the success of immunotherapy is not so obvious. Separately, it is necessary to mention rare histological types of kidney cancer, such as chromophobe and papillary cancer, for which generally less therapeutic possibilities are available compared to the clear cell variant of RCC. In most of the cases, these tumors are treated using the same strategy as clear cell RCC, but with less success [13].

The success of immunotherapy for RCC of various histological types largely depends on the expression of PD-L1 on tumor cells, which is a condition for its administration. It has also been shown that the success of immunotherapy is associated with the composition of the tumor microenvironment. It is now known that the tumor microenvironment plays a crucial role in the pathogenesis of the disease. The microenvironment is usually understood as tumor-associated cells of the inflammatory infiltrate, blood vessels, fibroblasts, extracellular matrix and, more recently, the resident tissue microbiome. In the case of some solid tumors, bacteria are an important component of their microenvironment.

Though the influence of the microbiome on tumor therapy, especially in the context of immunotherapy, is well documented [14,15], the mechanisms of this influence are not fully characterized. It has been shown that the composition of the microbiome can be considered as a potential clinical, diagnostic and prognostic marker in some tumors. For example, it has been shown that *P. gingivalis* in the esophageal mucosa can act as a biomarker for squamous cell carcinoma of this localization [16]. Tumor microbiome alpha diversity has been shown to be a predictor of outcome in patients with pancreatic adenocarcinoma after surgery [17]. The role of the resident microbiome has also been described for colorectal cancer (CRC). It was found that *F. nucleatum* has an immunosuppressive effect on the tumor microenvironment and may be a marker of poor prognosis for patients with CRC [18,19]. It is known that in prostate carcinoma, some types of resident bacteria inversely correlate with unfavorable clinical and morphological characteristics of patients and contribute to the immune response [20], while other types of microorganisms, on the contrary, form an immunosuppressive microenvironment and contribute to tumor progression [21]. For kidney cancer, the prognostic or therapeutic value of the tumor microbiome has not yet been clearly defined. Though, at present, none of the stromal tumor markers are used in routine clinical practice, accumulating evidence will surely lead to a change in this situation. Moreover, a number of recent studies have focused on the search for combinations of stromal markers, a comprehensive analysis of which will lead to an increase in their diagnostic and prognostic significance.

The presented study is focused on the analysis of the qualitative and quantitative composition of the microbiome of RCC tumors of various histological types, depending on their clinical and morphological characteristics and the phenotype of the inflammatory infiltrate of the tumor stroma. Analysis of the prognostic value of these individual markers and combinations thereof is performed.

## 2. Materials and Methods

### 2.1. Sample Collection and Ethics Statement

The samples were collected in accordance with the guidelines issued by the Ethics Committee of the N.N. Blokhin National Medical Research Center of Oncology. All patients gave written informed consent (available upon request). The study was performed in accordance with the principles outlined in the Declaration of Helsinki.

The study group included tumor samples in the form of paraffin blocks from 66 patients with RCC of various histotypes, obtained from patients who underwent examination and treatment at the N.N.Blokhin National Medical Research Center of Oncology of the Ministry of Health of Russia. Only samples from patients that did not receive any treatment prior to the surgery were included in the study. Eleven samples of conditionally normal kidney tissue were obtained from surgical kidney samples from patients that underwent surgery not related to RCC treatment. Out of all tissue samples, 30 RCC samples and 10 normal tissue samples were used for 16S rRNA sequencing. All (66 RCC and 11 conditionally normal) tissue samples collected were used for PCR and 66 RCC samples were used for immunohistochemical analysis. All procedures performed in the study involving patients and healthy donors complied with the ethical standards of the organization’s ethics committee and the 1964 Declaration of Helsinki and its subsequent amendments or comparable ethical standards. Informed consent was obtained from each of the participants included in the study. The clinical diagnosis in all patients was confirmed by the data of the morphological examination of the tumor according to the International Histological Classification of Kidney Tumors (WHO, 2016). A description of the studied 66 RCC samples is provided in Table 1.

### 2.2. Immunohistochemical Study

Formalin-fixed, paraffin-embedded RCC tissue samples were step-sectioned and stained with hematoxylin–eosin. Endogenous peroxidase activity was blocked with 3% hydrogen peroxidase for 10 min. HIER was provided in Tris–EDTA (pH 9.0) in a Decloaking Chamber (Biocare Medical, Pacheco, CA, USA). Sections were incubated with primary antibodies at room temperature: anti-CD68 (#61-0184 Genemed Biosciences, South San Francisco, CA, USA), anti-PU.1 (clone PBM-4G6, PrimeBioMed, Moscow, Russia); anti-CD163 (clone 10D6; Biocare Medical, Pacheco, CA, USA), CD56 (clone 123C3, Genemed Biosciences, South San Francisco, CA, USA) CD66b (SAB4301144, Sigma-Aldrich, St. Louis, MO, USA), CD20 (clone PBM-12F1; PrimeBioMed, Moscow, Russia), anti-iNOS (clone SP126; Sigma-Aldrich, St. Louis, MO, USA), anti-CD8 (clone C8/144B, Agilent, Santa Clara, CA, USA), anti-CD3 (#61-0011 Genemed Biosciences, South San Francisco, CA, USA) and anti-FOXP3 (clone D2W8E; Cell Signaling #98377). PowerStain 1.0 PolyHRP DAB kit (Genemed Biosciences, South San Francisco, CA, USA) was used for the detection.

To score the immunostaining results for macrophages (CD68, PU.1, CD163), T-cells (CD3, CD8, FoxP3), B-cells (CD20), NK cells (CD56) and neutrophils (CD66b), we randomly selected five representative high-power microscopic fields (×200 magnification) of the tumor sample per section and counted the numbers of positively stained cells (Olympus; Tokyo, Japan). Necrotic areas were ignored. The content of CD68, PU.1, CD163, CD3, CD8, FoxP3, CD20, CD56 and CD66b in tumor stroma was expressed as the median of the cell number in one microscopic field [22,23].

For iNOS, immunohistochemical staining was performed in tumor and stromal cells. The samples were divided into two groups depending on the presence of iNOS-positive cells in the stroma. Tumor staining was classified as positive when clear cytoplasmic staining was present in ≥1% of tumor cells for iNOS [24].

### 2.3. Quantitative PCR (qPCR)

Quantitative real-time PCR was performed to assess the abundance of the *16S* gene present in a subset of normal and tumor tissues. The PCR profile was as follows: 95 °C for 5 min, 40 cycles of 95 °C for 15 s, 55 °C for 30 s, 72 °C for 1 min. A total of 100 ng of extracted DNA and 0.5 μL of each primer (10 pmol) were added to 4 μL of the DFMasZGTaqMIX-2025 (Dialat, Moscow, Russia), and DNA-free water was added up to 20 μL total volume. All reactions were performed in triplicate for each sample. A negative control containing DNA-free water instead of DNA was used for each PCR run. The real-time qPCR data analysis was performed with the BioRad software Bio-Rad CFX Manager 3.1, (Hercules, CA, USA) with a manually set threshold. For the purposes of analysis, the metric was the number of cycles to cross the threshold (Ct value) as a measure of *16s* rRNA gene load and hence bacterial burden. A higher bacterial load resulted in a lower number of cycles to cross the threshold—that is, a lower Ct value [24,25].

### 2.4. 16S rRNA Gene Library Preparation and MiSeq Sequencing

DNA extraction from tissues was performed using the DNA FFPE kit (Qiagen, Hilden, Germany) according to the manufacturer’s instructions for capturing bacterial DNA. The quality of the extracted DNA was assessed with electrophoresis in 1% agarose gel and a Nanodrop 8000 (Thermo Fisher Scientific, Waltham, MA, USA). The DNA concentration was quantified using a Qubit 4.0 Fluorometer (Life Technologies, Carlsbad, CA, USA) with the dsDNA High-Sensitivity Assay Kit (Life Technologies, Carlsbad, CA, USA).

Preparation of the DNA libraries was performed according to the Illumina protocol (Part #15044223, Rev. B.) with primers targeting the V3–V4 regions of the SSU ribosomal RNA (rRNA) gene, S-D-Bact-0341-b-S-17 (5′-CCTACGGGNGGCWGCAG-3′) as the forward primer and S-D-Bact-0785-a-A-21 (5′-GACTACHVGGGTATCTAATCC-3′) as the reverse primer [26]. The reaction mixture (10 µL) contained both primers, 0.1 µM each; 80 µM dNTPs; 0.2 U Q5 High-Fidelity DNA Polymerase. The following PCR program was used: 95 °C for 3 min, 40 cycles 95 °C for 30 s, 56 °C for 30 s, 72 °C for 30 s, final extension 72 °C for 5 min. For each reaction, three replicates were amplified. Then, the replicates were mixed together and cleaned using Agencourt AMPure XP beads (Beckman Coulter, Brea, CA, USA). Paired-end 2 × 250 bp sequencing was performed on the MiSeq platform (Illumina, San Diego, CA, USA) with the Reagent Kit v.2 (Illumina, San Diego, CA, USA).

DNA library preparation, sequencing and bioinformatics treatment were performed in the Center of Shared Scientific Equipment “Persistence of Microorganisms” of the Institute for Cellular and Intracellular Symbiosis UrB RAS, Russia.

### 2.5. Bioinformatics Treatment

At the first stage, the raw reads obtained as a result of sequencing were evaluated with FastQC v. 0.11.7. Evaluation was necessary to determine the parameters of further processing and included an assessment of the quality and length of reads and presence of adapter sequences. Paired-end reads were merged with a maximum number of mismatches of 10 using Usearch v. 11.0.667 [27,28]. Adapter sequences were removed with *Trimmomatic* v 0.36 [29,30]. After merging and adapter removal, the reads were re-evaluated with FastQC v. 0.11.7. Subsequent treatment of merged reads was conducted with Usearch v. 11.0.667 [27,28] and included quality filtering (expected error or maximum less than 1.00) and amplicon size selection (420-bp minimal size). Evaluation of the filtering quality was carried out with FastQC v 0.11.7. The next stage included dereplication and clustering of the filtered reads. As a result of dereplication and clustering, operational taxonomic units (OTUs) were formed. Chimeric sequences were detected and removed using the UCHIME2 algorithm [28]. Final OTUs were aligned to the initial merged reads using global alignment (usearch_global tool) at a 97% level of similarity. As a result of global alignment, the number of merged reads corresponding to every OTU was estimated. Contaminant OTUs were identified and removed via the usearch_ublast command by matching the sequences of trial samples and negative control samples. The taxonomic classification of sequences was conducted using the RDP reference database [31,32]. For OTUs with taxonomic position estimated at a low level of support (ab_score less than 0.7), taxonomy was determined using the NCBI database [33]. OTUs identified as host (human) were removed from the dataset.

Heatmap and clustering analyses were performed at the taxonomic level of bacterial genera using MicrobiomeAnalyst with the following parameters: Euclidian distance measure and Ward clustering algorithm [34]. Differential abundance of bacterial genera was evaluated using the limma method [35]. The genera having log2fold > 1 and FDR-corrected *p*-value < 0.05 were considered significantly different in terms of differential abundance. Volcano plot was drawn with the Bioconductor EnhancedVolcano package [36].

### 2.6. Availability of Data

Raw sequence data and metadata are available at the NCBI BioProject database under project ID PRJNA838259.

### 2.7. Statistical Analyses

The diversity of microbiomes within samples (alpha diversity) was evaluated with indices Chao1 and Shannon. Similarity of microbiomes between samples (beta diversity) was assessed using the Bray–Curtis distance. To visualize the similarity of microbiomes between samples, Principal Coordinates Analysis (PCoA) was performed. Taxa that were significantly different between different tissues were identified with MicrobiomeAnalyst [37], developed for microbiome statistics applications. Differences in the overall microbial composition between different groups were assessed by the Mann–Whitney nonparametric test.

IHC statistical analysis was performed using GraphPad Prism ver. 9 by GraphPad Software. The Spearman rank correlation coefficient was used to compare between groups to examine the association between immune marker expression and clinicopathological characteristics and bacterial burden. Continuous variables were compared between groups by the Mann–Whitney nonparametric test. Survival length was determined as the time period from the date of surgery to the date of death or the last clinical attendance. Survival curves were derived using the Kaplan–Meier method, and differences between curves were analyzed using the log-rank test. To assess the potential impact of various risk factors on survival, a multivariate analysis was additionally performed using a nonparametric Cox proportional hazards model. Differences and correlations were considered statistically significant at *p* < 0.05.

## 3. Results

### 3.1. Renal Tissue Microbiome

To analyze the composition of the microbial community using 16S rRNA sequencing, 30 RCC samples (10 samples of clear cell RCC, 10 samples of papillary RCC and 10 samples of chromophobe RCC) out of 66 collected were used. No specific inclusion criteria were used for the selection of these 30 RCC samples. Nineteen RCC samples were from patients with stage I–II disease and 11 samples were from patients with stage III–IV disease. Additionally, 10 samples of normal kidney tissue out of 11 collected were analyzed using 16S rRNA sequencing.

Analysis of the taxonomic composition of the microbial community of renal and RCC tissues revealed the presence of 14 phyla (Table 2) and 170 genera. The predominant types of microorganisms found in both tumors and samples of normal kidney tissue were *Actinobacteria*, *Proteobacteria*, *Firmicutes*, *Cyanobacteria_Chloroplast* and *Bacteroidetes*.

**Figure 1:**
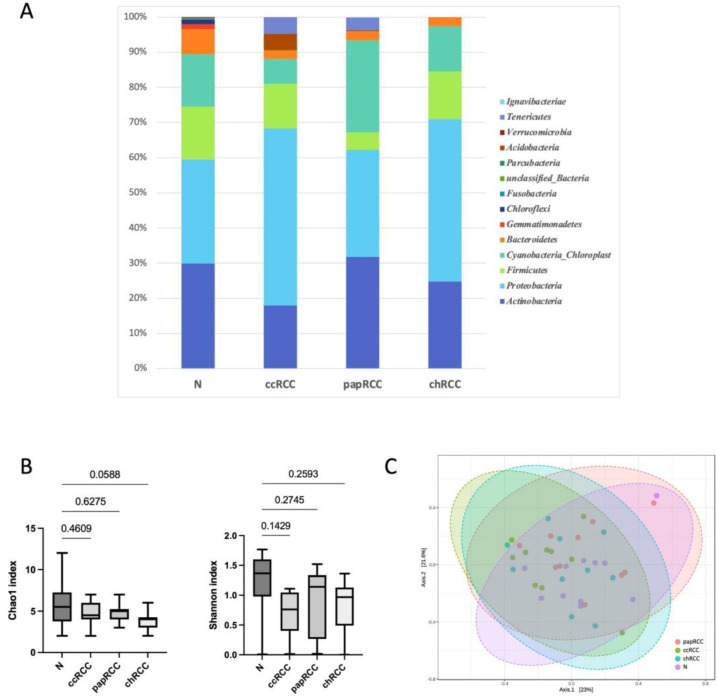
Taxonomic composition of the microbial community of renal tissues on the level of phylum. (**A**) Characterization of renal cell cancer (RCC) microbiota. Colored lines represent relative abundance at the phylum level for tumor (ccRCC, papRCC, chRCC) and normal tissue (N) samples. (**B**) Taxonomic α-diversity calculated with Shannon and Chao1 indices between N and T groups; *p*-values are indicated above the lines. (**C**) PCoA plot based on Bray–Curtis distance of renal microbiome between tumor and normal tissues (PERMANOVA). F-value: 0.97931; R-squared: 0.073562; *p*-value < 0.462.

It should be noted that even at the level of phylum, there were differences between normal kidney tissue and kidney tumors of different histotypes. For example, bacteria of the *Tenericutes* phylum were present in ccRCC and papRCC tumors and were absent in normal tissue and chromophobe tumors. It should also be noted that bacteria of the *Gemmatimonadetes*, *Chloroflexi*, *Fusobacteria*, *Parcubacteria* and *Verrucomicrobia* phyla were found only in samples of normal kidney tissue. Next, an analysis of taxonomic diversity in the studied samples was carried out. The results are presented in Figure 1.

Despite the fact that no significant differences for taxonomic alpha and beta diversity were observed between tumor and normal tissue (Shannon and Chao1 indices) at the phylum level, it is worth noting that more taxonomic diversity was observed in samples of normal tissue compared to tumor tissue. The smallest number of bacterial phyla was found in samples of chromophobe RCC.

Next, we analyzed the relative abundance of bacteria at the genus level in the tumor and normal tissue.

For further analysis, genera of bacteria with an abundance level of more than 0.1% were taken into account. There were 113 such dominant genera, and the most pronounced ones are presented in Table 3.

The most represented genera identified in normal kidney tissue samples were *Kocuria*, *Phyllobacterium*, *Micrococcus*, *Cutibacterium*, *Corynebacterium*, *Rothia*, *Streptococcus* and *Acinetobacter*. For tumor tissue samples, the most represented genera were *Cutibacterium*, *Sphingomonas*, *Roseomonas*, *Staphylococcus*, *Mesomycoplasma*, *Massilia*, *Escherichia_Shigella* and *Photobacterium* for clear cell RCC; for papillary cancer, they were *Cutibacterium*, *Corynebacterium*, *Escherichia_Shigella*, *Clavibacter*, *Enhydrobacter*, *Phyllobacterium*, *Mesomycoplasma*, *Simplicispira*, and for chromophobe cancer, they were *Escherichia_Shigella*, *Novosphingobium*, *Cutibacterium*, *Psychrobacter*, *Lactococcus*, *Acinetobacter*, *Jeotgalicoccus* and *Corynebacterium.* This indicates that the taxonomic composition of the microbiome in kidney tumors of different histotypes varies. The heatmap and clustering analysis, however, did not find bacterial genera associated with a certain group of cancer samples or normal tissue samples (Supplementary Figure S1). Moreover, the heatmap demonstrated neither a clear pattern of the occurrence and relative abundance of the bacterial genera in both cancer and normal tissue samples, nor an association of certain taxa with a certain group of samples. Limma analysis of the differential abundance of bacterial genera with volcano plot building showed no genera with statistically significant FDR-corrected *p*-value *p* < 0.05 (Supplementary Figure S2).

**Figure 2:**
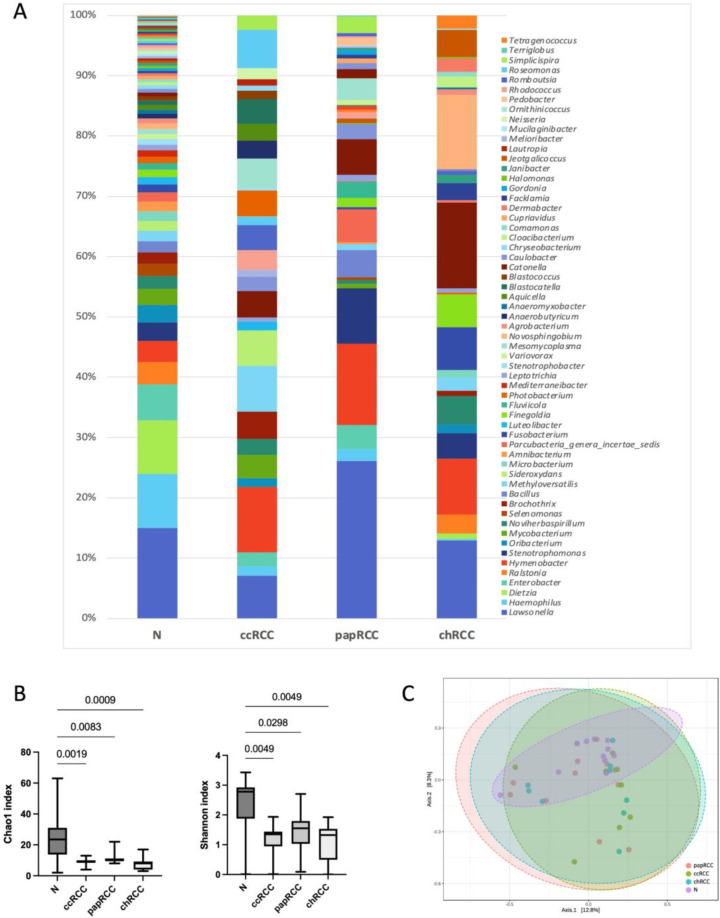
Taxonomic composition of the microbial community of renal tissues on the level of genus. (**A**) Characterization of renal cell cancer (RCC) microbiota. Colored lines represent relative abundance at the genus level for tumor (ccRCC, papRCC, chRCC) and normal tissue (N) samples. (**B**) Taxonomic α-diversity calculated with Shannon and Chao1 indices between N and tumor groups. *p*-values are indicated above the lines. (**C**) PCoA plot based on Bray–Curtis distance of renal microbiome between tumor and normal tissues (PERMANOVA). F-value: 1.1096; R-squared: 0.082541; *p*-value < 0.226.

We also analyzed alpha and beta diversity in each group at the level of bacterial genera. The results are presented in Figure 2.

The analysis revealed significant differences in alpha diversity between samples of normal and tumor kidney tissue in all histological types of RCC. It should be noted that a significant decrease in the number of detected taxa was observed for tumors of all types. To evaluate the similarities of all samples, ecologic Bray–Curtis/unweighted UniFrac distances were calculated and visualized by PCoA plot. There were no significant differences between the tumor and normal tissue groups at genera level.

**Figure 3:**
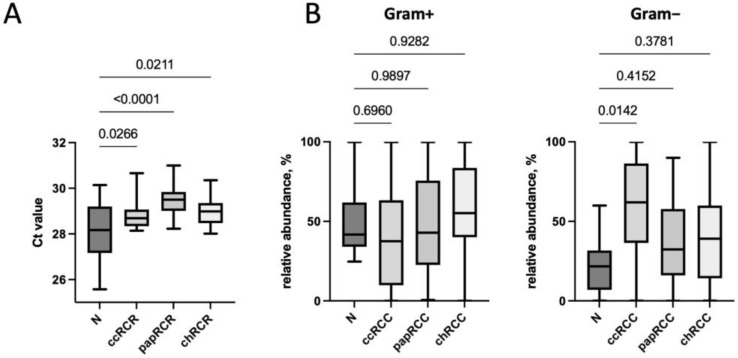
Total bacterial load (**A**), as well as the relative content of Gram-positive and Gram-negative bacteria (**B**), depending on the histological type of the tumor. *p*-values are indicated above the lines.

Next, we performed a quantitative analysis of bacteria in the tumor tissue compared to conditionally normal kidney tissue by real-time PCR. It was found that the total bacterial load in normal kidney tissue was higher than in the tumor tissue (Figure 3A). It should be noted that of all studied histological types of RCC, the smallest bacterial load was found in papillary RCC samples. The analysis of the relative content of Gram-positive and Gram-negative microorganisms was also carried out. In general, the predominance of Gram-positive microorganisms over Gram-negative ones was observed in the microbiome of both normal tissue and some types of tumors. Only for the clear cell type of RCC, the predominance of Gram-negative bacteria was found (Figure 3B).

**Figure 4:**
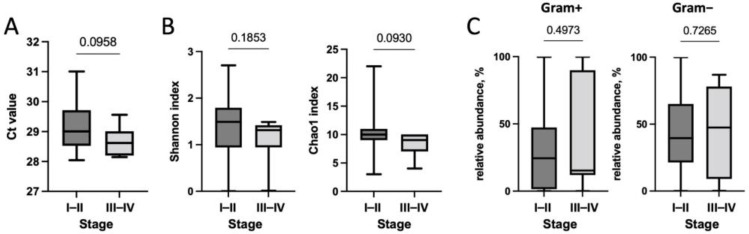
Analysis of the association of the qualitative and quantitative composition of the microbiome ((**A**)—total bacterial load, (**B**)—Shannon and Chao1 indexes) with the RCC stage. (**C**)—association of relative content of Gram-positive and Gram-negative bacteria with the RCC stage. *p*-values are indicated above the lines.

Next, we studied the changes in the microbiome during RCC progression—namely, its qualitative and quantitative composition depending on the stage of the disease. The results are presented in Figure 4.

We established that tumors of early and late stages did not differ significantly in terms of total bacterial load and taxonomic diversity; however, with an increase in the stage of RCC, a trend towards a decrease in these indicators was observed. Moreover, no changes in the content of Gram-positive and Gram-negative bacteria, depending on the stage of the disease, were observed.

### 3.2. Renal Tissue Stroma

**Figure 5:**
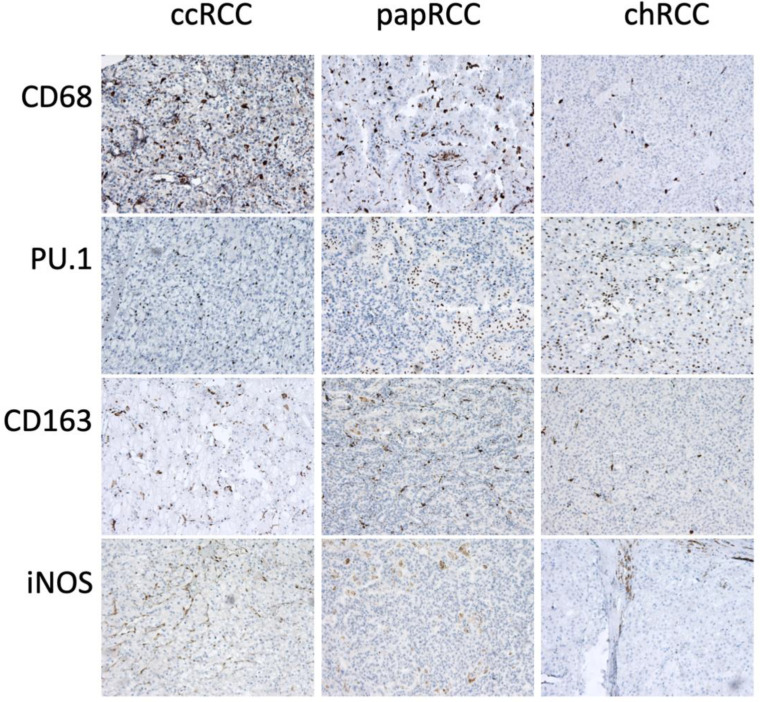
Immunohistochemical staining of RCC samples for macrophage markers CD68, PU.1, CD163 and iNOS. Magnification is 400×.

**Figure 6:**
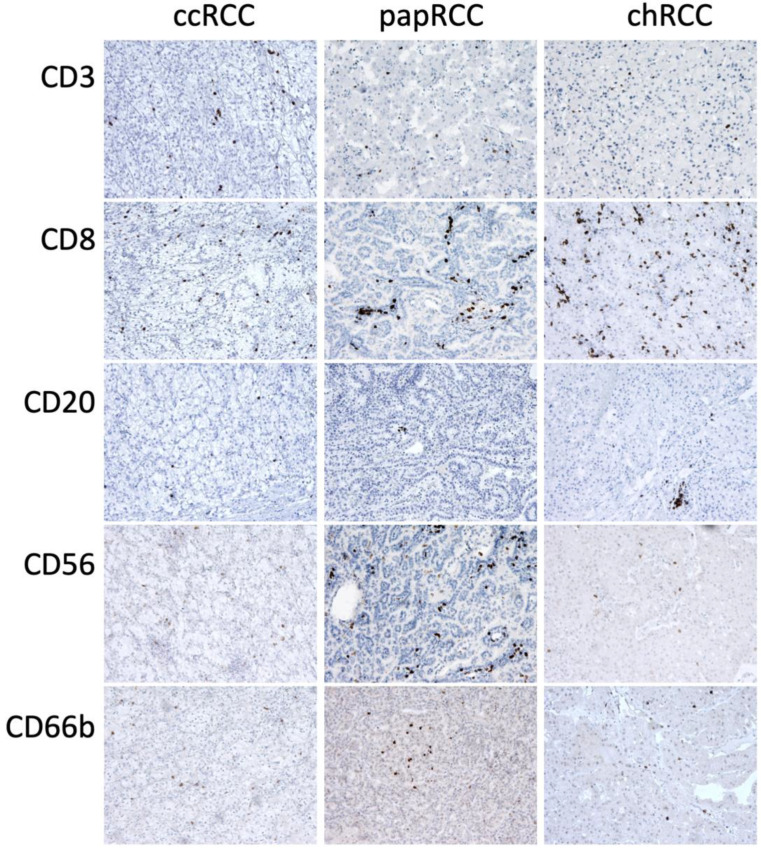
Immunohistochemical staining of RCC samples for CD3, CD8, CD20, CD56 and CD66b. Magnification is 400×.

Phenotyping of the cells of the inflammatory infiltrate of the tumor stroma was carried out using immunohistochemistry. Macrophages of two main phenotypes, M1 and M2, were studied using CD68 and PU.1 markers as pan-macrophage markers: CD163 to detect the M2 phenotype and iNOS to detect the M1 phenotype (Figure 5). T-cells were detected using CD3, CD8 and FoxP3, NK cells using CD56 and neutrophils using CD66b (Figure 6).

Analysis of the association of immune cell content in the tumor stroma with the clinical and morphological characteristics of patients was performed (Table 4).

We established that the phenotype of the inflammatory infiltrate of tumor stroma cells is not associated with the stage of the disease but differs depending on the tumor histotype (Table 4). For macrophages, neutrophils and T-cells, a significant decrease was found in chromophobe RCC. Moreover, in general, the content of all stromal cells in chromophobe tumors was found to be lower compared to other histological types of kidney cancer (Table 4). Alpha diversity analysis also showed no significant differences between groups with high and low tumor stromal cell infiltration (data not shown).

iNOS was used to detect cytotoxic macrophages (M1). For iNOS, a qualitative assessment was carried out by the presence of its expression in macrophages of the tumor stroma. Of the 66 tumor tissue samples examined, nine were iNOS-positive. The analysis of iNOS content among tumors of different histotypes showed that it was more often found in the stroma of papillary cancer (26% of cases), while iNOS+ cells were detected in samples of chromophobe cancer only in 4% of cases.

Next, a correlation analysis of the total bacterial load with the tumor stroma phenotype was carried out. The data are presented in Table 5.

As can be seen from the presented results, a significant correlation between the level of bacterial load and the phenotype of the tumor stroma was observed for PU.1 (r = 0.301, *p* = 0.013) and CD66b (r = 0.326, *p* = 0.007). The group of tumors with high content of PU.1- and CD66b-positive cells in the stroma was characterized by a lower bacterial load in general (Table 5). Next, we analyzed the correlation of the total bacterial load with the phenotype of tumor stroma cells for each of the studied histological tumor types. No such correlations were found for chromophobe and papillary RCC (Table 5). For the clear cell carcinoma group, a significant inverse correlation was found between the total bacterial load and the number of CD20+ and CD8+ cells in the stroma (r = −0.734, *p* = 0.019 and r = −0.681, *p* = 0.035, respectively).

A correlation analysis was also carried out for the phenotype of macrophages of the tumor stroma and certain genera of bacteria. We found that in the group of tumors characterized by high content of PU.1+ cells, as well as CD163+ cells (M2 macrophages), there was an inverse correlation with the content of bacteria of the genera *Phocaeicola*, *Brevundimonas* and *Acinetobacter* (r = −0.354, *p* = 0.055; r = −0.394, *p* = 0.031; r = −0.503, *p* = 0.005 respectively). In the group of tumors characterized by high content of M1 macrophages (iNOS +), there was a direct correlation with bacteria of the genera *Phyllobacterium*, *Mesomycoplasma* and *Staphylococcus* (r = −0.371, *p* = 0.044; r = −0.386, *p* = 0.035 and r = 0.418, *p* = 0.021).

### 3.3. Survival Analysis

**Figure 7:**
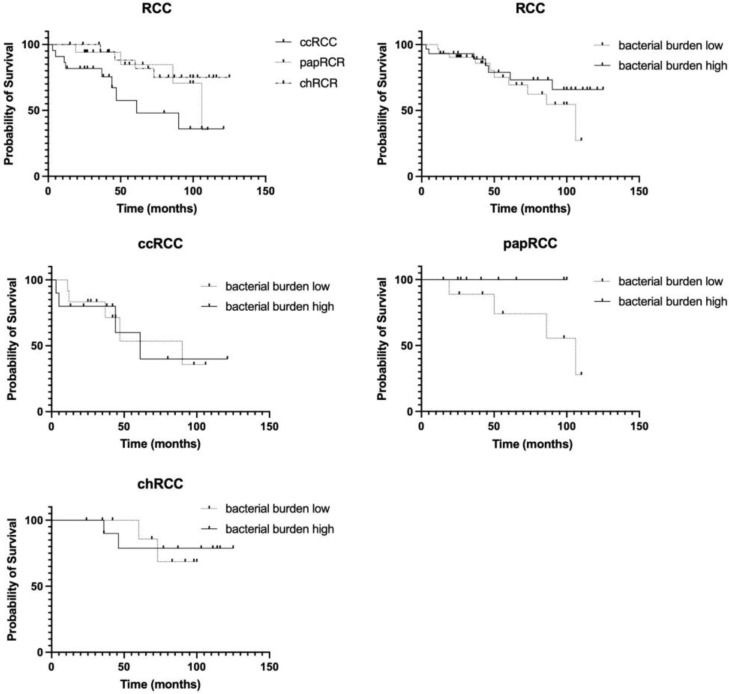
Prognostic significance of the histological type of the tumor in general, as well as the total bacterial load, in RCC.

**Figure 8:**
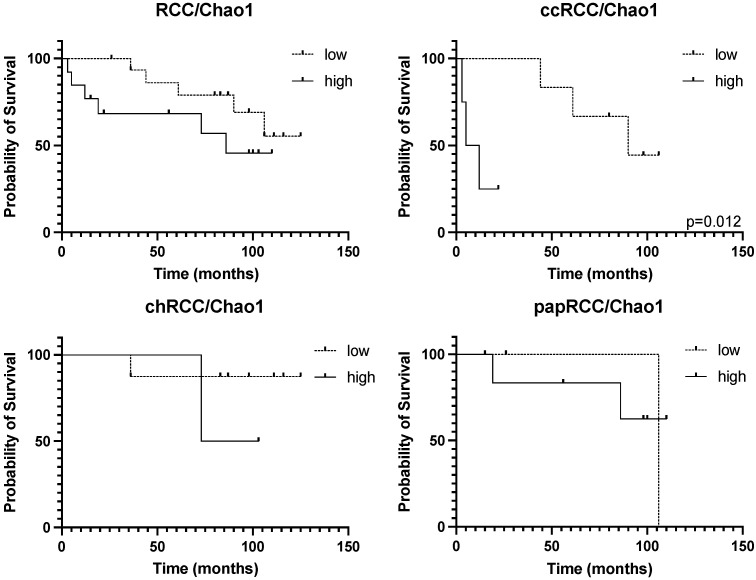
Prognostic significance of taxonomic diversity for RCC of different histotypes.

At the last stage of the study, we analyzed the prognostic significance of the expression of all the studied stromal markers, as well as their combinations. The results are presented in Figure 7 and Figure 8.

The analysis performed showed significant prognostic differences between different histological types of RCC (Figure 7). As expected, the most prognostically unfavorable type of tumor is clear cell carcinoma, while the other two histotypes studied have a more favorable prognosis. Analysis of the overall bacterial load in the RCC group as a whole did not reveal its prognostic significance (Figure 7, upper right panel). Next, we analyzed the prognostic significance of the bacterial load depending on the histological type of the tumor. We found that only papillary tumors with a high bacterial load show a tendency towards a favorable prognosis. It is important to mention that this histological type of RCC is characterized by the lowest bacterial load compared to other histotypes of RCC (Figure 7).

Since different histological types of RCC are characterized by different numbers of taxa of resident microorganisms, and the smallest number of them is detected in cases of chromophobe kidney cancer, an analysis of the prognostic significance of the presented taxa both for RCC in general and for histological groups separately was carried out.

The prognostic significance of the number of taxa of microorganisms was observed only for the clear cell RCC (Figure 8, upper right panel). For kidney cancer in general, a similar trend was observed, which may have been due to the group of clear cell tumors (Figure 8).

Next, we analyzed the prognostic significance of stromal markers of kidney tumors in general and for various tumor histotypes. Statistical analysis of the overall survival of patients with RCC is presented in Table 6.

We found prognostic significance of the CD68, CD163 and CD8-positive cell number in the tumor stroma. The high content of these markers in the tumor stroma is an unfavorable prognostic factor (Table 6). At the same time, iNOS+ macrophages in the tumor stroma and CD20+ T-cells showed favorable prognostic value in RCC (Table 6).

**Figure 9:**
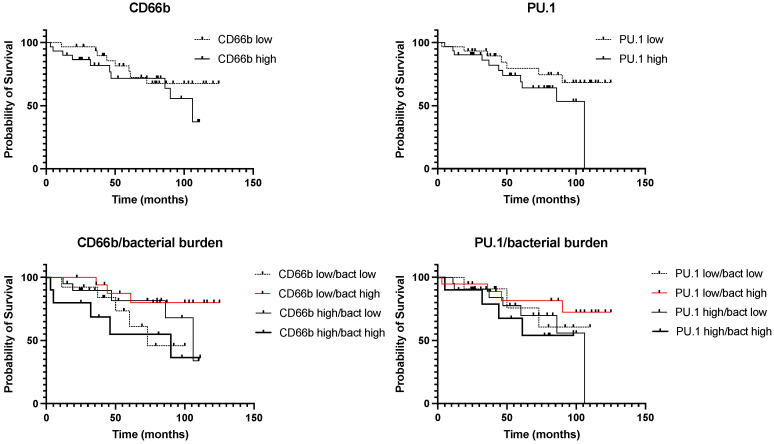
Prognostic significance of the total bacterial load and cell phenotype of the tumor infiltrate.

Next, we analyzed the complex prognostic significance of the total bacterial load with the phenotype of tumor infiltrate cells. The results are presented in Figure 9.

Although we found a correlation between the content of PU.1 and CD66b in tumors and the total bacterial load (Table 6), the analysis of survival depending on the content of these stromal markers and the total bacterial load did not reveal any correlation (Figure 9, upper panels). Therefore, we conducted a comprehensive analysis of the prognostic significance of PU.1 and CD66b with the total bacterial load. We found that the group of patients characterized by CD66b high/bacteria high had an extremely unfavorable prognosis compared with the group of CD66b low/bacteria high (HR 4.046; *p* = 0.037) (Figure 9). Similar results were obtained for PU.1; however, the data did not reach statistical significance (Figure 9). The obtained results indicate that for kidney cancer, the expression of some stromal markers is prognostically significant depending on the bacterial load.

## 4. Discussion

It is known that the microenvironment plays a significant role in the pathogenesis of RCC and is involved in the disease initiation, progression and response to treatment. However, the qualitative and quantitative composition of the tumor microenvironment is highly dependent on the histological type of the tumor, and its role in the context of disease progression has not yet been determined [38].

The main cells of the immune infiltrate of kidney tumors are macrophages, T-cells and neutrophils. It is known that CD68 itself can be a marker of poor prognosis of RCC, which is consistent with our data [39]. In contrast, cytotoxic macrophages (M1) are often associated with a favorable prognosis in various types of solid tumors [40]. In the case of RCC, the prognostic role of M1 macrophages has not been studied to date. We here established that a high content of M1 macrophages is a favorable prognostic factor for RCC.

Less research has been devoted to tumor-associated neutrophils in the case of RCC compared to macrophages. From the literature data, it is known that tumor-associated neutrophils contribute to the progression of the disease and act as a marker of the worst prognosis for patients with oncological diseases of various localizations [41]. There is evidence that an increased number of CD66b+ cells in tumor tissue is associated with worse prognosis in patients with primary and metastatic renal cell carcinoma [42,43]. Moreover, CD66b+ neutrophil density has been shown to be increased in the tumor nodules compared to adjacent normal tissue and is associated with disease progression [44]. CD66b+ can also be used as a predictive marker for the response of patients with RCC to immunotherapy. For example, it was shown that increased content of CD66b+ neutrophils in tumor tissue is a predictor of a poor response to therapy with tyrosine kinase inhibitors [45]. The data presented by us in this study showed that stromal CD66b+ is not a prognostic factor of RCC and it did not correlate with the stage of the disease. However, it should be noted that the samples of papillary RCC were characterized by high content of cells of this type compared to other histological types of RCC—in contrast, for example, to CD68+ cells, the largest number of which was noted in samples of clear cell carcinoma. The presence of papillary RCC samples in the study group may have influenced the prognostic significance of CD66b+ cells, since these tumors usually have better prognosis than ccRCC.

Until recently, normal kidney tissue was thought to be sterile and free of microorganisms. However, it has been found that in some diseases, bacteria can enter the kidney tissue through the bloodstream [46]. The resident microbiome of kidney tumors is described rather poorly. There are several works in the literature [47,48,49] describing the analysis of the resident tissue microbiome of kidney tumors, and most of them are focused on the clear cell RCC histotype only. In the present study, we analyzed the resident microbiome of kidney tumors of various histotypes for the first time. We established that the predominant phyla of microorganisms found in both tumor and normal kidney tissue were *Actinobacteria*, *Proteobacteria*, *Firmicutes*, *Cyanobacteria_Chloroplast* and *Bacteroidetes*, which is consistent with other studies [49]. We also found that the tumor tissue microbiome has lower alpha diversity, compared to the adjacent conventional normal kidney tissue. Our study showed a significant decrease in alpha diversity indices in all histological types of RCC compared with normal kidney tissue. Since tissues of a normal kidney, rather than adjacent normal tissue from the patients with kidney tumors, were used as control samples in this study, the differences that we detected were more pronounced.

The analysis of the dominant genera of the microbiome did not reveal significant differences in the studied groups; however, it should be noted that different histological types of kidney tumors differed significantly in their qualitative taxonomic composition. Studies on the taxonomic diversity of various histological types of kidney tumors have not been described in the literature so far.

Previously, we have shown that in the case of lung and esophageal cancer, a high bacterial load in the tumor, combined with increased expression of iNOS, is a favorable prognostic factor, and a high bacterial load, combined with local immunosuppression, on the contrary, is a marker of poor prognosis [23,24]. Here, we demonstrate that for kidney cancer, iNOS is an independent marker of a favorable prognosis. However, it should be noted that its expression was observed only in 9 out of 66 samples studied by us; therefore, it was not possible to conduct an analysis of iNOS in combination with the level of bacterial load due to the small size of the comparison groups.

The main limitation of the study is the size of the sample, which was limited primarily by the rare histotypes of RCC—papillary and chromophobe ones. This limited sample size is one of the possible reasons that no specific differences in bacterial genera were found.

In this work, a significant correlation was found between the level of bacterial load and the content of PU.1+ and CD66b+ stromal cells. The group of tumors with high content of PU.1+ cells and CD66b+ cells in the stroma was characterized by a lower bacterial load. Therefore, at the next stage of the work, an analysis of the prognostic significance of these markers was carried out. We demonstrated here that the number of PU.1+ cells and CD66b+ cells have prognostic significance only in the group of tumors with a high bacterial load, where they are associated with a poor prognosis. Since macrophages and neutrophils are the main cell types responsible for recognizing a pathogen and responding to a bacterial infection, and, in tumor conditions, they are not able to show their antitumor activity due to the induction of tolerance by low concentrations of bacteria, their high content becomes a factor associated with an unfavorable prognosis.

In conclusion, for the first time, an extensive study of the resident tumor microbiome was carried out together with the analysis of the phenotype of the inflammatory infiltrate of stromal cells of various histological types of kidney cancer. Identified correlations indicate the great potential value of the tumor microbiome in combination with the properties of stromal cells as prognostic markers. Further research on larger cohorts will allow the discovery of the role of the tumor microbiome and even specific bacteria in RCC pathogenesis. Microbiome features combined with tumor stroma analysis will allow for the development of novel diagnostic tests and personalized therapeutic strategies in patients with kidney tumors.

## Figures and Tables

**Table 1:** Clinical and morphological characteristics of RCC patients.

Characteristic	Number of Cases (%)
Age	
≤58	34 (52%)
>58	32 (48%)
Gender	
Male	34 (52%)
Female	32 (48%)
Histology	
ccRCR	23 (35%)
papRCC	19 (29%)
chRCR	24 (36%)
Stage	
I-II	48 (73%)
III-IV	18 (27%)
Tumor size	
T1-T2	50 (76%)
T3-T4	16 (24%)
Nodal status	
N0	63 (95%)
N+	3 (5%)

**Table 2:** Taxonomic composition of kidney tumors at the level of phylum.

Phylum	N	ccRCC	papRCC	chRCC	*p*-Value
*Actinobacteria*	29.88	18.00	31.77	24.79	0.7995
*Proteobacteria*	29.59	50.31	30.49	46.11	0.3673
*Firmicutes*	15.04	12.75	4.99	13.60	0.7174
*Cyanobacteria_Chloroplast*	15.00	7.11	26.08	12.96	0.4798
*Bacteroidetes*	7.11	2.28	2.68	2.52	0.6439
*Gemmatimonadetes*	1.32	0.00	0.00	0.00	0.0700
*Chloroflexi*	1.24	0.00	0.00	0.00	0.1629
*Fusobacteria*	0.20	0.00	0.00	0.00	0.1073
*unclassified_Bacteria*	0.18	0.14	0.00	0.00	0.5198
*Parcubacteria*	0.15	0.00	0.00	0.00	0.4040
*Acidobacteria*	0.12	4.59	0.17	0.00	0.5429
*Verrucomicrobia*	0.12	0.00	0.00	0.00	0.4040
*Tenericutes*	0.02	4.82	3.62	0.00	0.5654
*Ignavibacteriae*	0.00	0.00	0.22	0.00	0.4040

**Table 3:** Taxonomic composition of kidney tumors at the genus level.

Genus	N	ccRCC	papRCC	chRCC	*p*-Value
*Kocuria*	8.93	0.00	0.00	0.95	0.3949
*Phyllobacterium*	5.92	2.25	3.97	0.00	0.3311
*Micrococcus*	3.67	0.00	0.00	3.15	0.4231
*Cutibacterium*	3.55	10.90	13.52	9.22	0.5331
*Corynebacterium*	3.07	0.00	9.16	4.19	0.2990
*Rothia*	2.93	1.46	0.00	1.47	0.2475
*Streptococcus*	2.67	3.86	0.73	0.00	0.7523
*Acinetobacter*	2.13	2.64	0.62	4.75	0.7671
*Nocardioides*	1.95	0.00	0.51	0.00	0.3232
*Massilia*	1.95	4.54	0.00	0.87	0.6030
*Enhydrobacter*	1.80	0.00	4.50	0.00	0.3910
*Sphingomonas*	1.80	7.57	1.00	2.24	0.3585
*Staphylococcus*	1.60	5.91	0.00	0.00	0.4409
*Lactobacillus*	1.60	0.00	0.00	1.17	0.4023
*Fibrella*	1.58	0.00	0.29	0.00	0.4727
*Clavibacter*	1.53	0.00	5.41	0.00	0.4462
*Psychrobacter*	1.30	0.00	0.38	7.14	0.5743
*Flavobacterium*	1.26	1.45	0.00	0.00	0.5244
*Lactococcus*	1.19	0.00	1.53	5.44	0.3018
*Croceibacterium*	1.14	0.00	2.69	0.00	0.4798
*Phocaeicola*	1.07	0.00	0.12	0.29	0.6109
*Methylobacterium*	1.04	0.00	0.00	0.00	0.4040
*Brevundimonas*	0.94	0.66	1.01	0.69	0.8674
*Escherichia_Shigella*	0.65	4.34	5.95	14.28	0.7391
*Pseudomonas*	0.63	2.42	2.51	0.00	0.6729
*Brachybacterium*	0.42	0.00	0.00	2.77	0.4619
*Aureimonas*	0.41	0.00	0.00	1.28	0.4733
*Burkholderia*	0.38	1.14	0.06	0.00	0.4501
*Paracoccus*	0.36	3.24	1.02	0.00	0.3761
*Lawsonella*	0.35	4.15	0.00	0.66	0.5178
*Haemophilus*	0.35	1.37	0.00	0.00	0.4667
*Photobacterium*	0.10	4.29	0.00	0.00	0.4203
*Mesomycoplasma*	0.02	4.82	3.62	0.00	0.5654
*Novosphingobium*	0.02	0.00	0.00	12.35	0.1438
*Anaerobutyricum*	0.00	2.97	0.00	0.00	0.4040
*Aquicella*	0.00	2.75	0.00	0.00	0.4094
*Blastocatella*	0.00	4.15	0.00	0.00	0.4034
*Blastococcus*	0.00	1.38	0.00	0.00	0.4040
*Catonella*	0.00	0.00	1.55	0.00	0.4042
*Caulobacter*	0.00	0.00	1.00	0.00	0.4040
*Cloacibacterium*	0.00	0.00	0.00	1.84	0.4041
*Dermabacter*	0.00	0.00	0.00	2.18	0.4042
*Jeotgalicoccus*	0.00	0.00	0.00	4.50	0.4041
*Lautropia*	0.00	1.10	0.00	0.00	0.4043
*Neisseria*	0.00	1.86	0.00	0.00	0.4042
*Pedobacter*	0.00	0.00	1.32	0.00	0.4040
*Roseomonas*	0.00	6.33	0.00	0.07	0.4100
*Simplicispira*	0.00	2.40	2.75	0.00	0.5740
*Tetragenococcus*	0.00	0.00	0.00	2.20	0.4044

**Table 4:** Association of the content of CD68, PU.1, CD163, CD66b, CD56, CD20, CD3, CD8, FoxP3 positive cells in the stroma with the histological type of tumor and stage of the disease (median 25–75%).

	Histology				Stage		
	ccRCC	papRCC	chRCC	*p*	I–II	III–IV	*p*
CD68	18.0 (8.6–22.4)	11.4 (5.8–15.4)	9.5 (4.5–13.4)	0.041 *	10.8 (6.1–16.8)	18.5 (4.9–22.5)	0.230
PU.1	17.4 (7.2–25.8)	20.2 (7.8–29.8)	7.0 (3.6–14.6)	0.054	10.8 (5.3–24.7)	17.3 (6.7–27.7)	0.268
CD163	11.6 (6.2–15.8)	8.4 (5.6–11.0)	3.6 (2.7–6.8)	0.043 *	6.7 (3.3–11.5)	9.9 (3.8–15.7)	0.203
CD66b	2.6 (1.4–5.6)	4.8 (2.0–8.0)	1.7 (1.2–3.0)	0.019 *	2.5 (1.2–5.2)	2.3 (1.6–5.0)	0.980
CD56	1.8 (0.6–11.8)	1.2 (0.8–3.6)	1.7 (0.7–9.5)	0.611	1.6 (0.7–7.3)	1.5 (0.9–9.85)	0.788
CD20	2.6 (1.0–5.8)	3.2 (1.4–5.4)	2.7 (0.6–6.5)	0.603	3.0 (0.9–6.0)	3.6 (0.9–4.9)	0.957
CD3	16.8 (4.2–32.8)	7.2 (5.4–12.4)	1.8 (1.1–6.1)	0.001 *	5.5 (1.9–12.6)	9.7 (1.8–34.3)	0.152
CD8	7.4 (1.8–20.0)	7.0 (2.0–10.6)	2.4 (0.6–3.6)	0.002 *	3.5 (1.5–7.7)	8.1 (1.2–22.75)	0.141
FoxP3	0.2 (0.0–0.6)	0.4 (0.0–0.8)	0.4 (0.0–0.8)	0.383	0.4 (0.0–0.8)	0.2 (0.0–0.6)	0.336

* indicates *p*-values of statistically significant differences.

**Table 5:** Correlation analysis of the total bacterial load with the phenotype of the tumor stroma.

	Bacterial Burden vs. CD68	Bacterial Burden vs. PU.1	Bacterial Burden vs. CD163	Bacterial Burden vs. CD66b	Bacterial Burden vs. CD56
**Spearman r**					
r	0.040	0.301	0.130	0.326	0.056
95% confidence interval	−0.2107 to 0.2864	0.05736 to 0.5123	−0.1226 to 0.3672	0.08423 to 0.5320	−0.1949 to 0.3013
***p*-value**					
*p* (two-tailed)	0.747	0.013 *	0.297	0.007 *	0.651
	**Bacterial Burden** **vs.** **iNOS**	**Bacterial Burden** **vs.** **CD20**	**Bacterial Burden** **vs.** **CD3**	**Bacterial Burden** **vs.** **CD8**	**Bacterial Burden** **vs.** **FoxP3**
**Spearman r**					
r	0.124	0.161	0.172	0.100	0.186
95% confidence interval	−0.1289 to 0.3617	−0.09073 to 0.3948	−0.07977 to 0.4041	−0.1524 to 0.3407	−0.06501 to 0.4164
***p*-value**					
*p* (two-tailed)	0.321	0.194	0.165	0.423	0.132

* indicates *p*-values of statistically significant differences.

**Table 6:** Statistical analysis of prognostic significance stromal markers in patients with RCC.

Markers	Univariate Analysis			Multivariate Analysis		
	HR	95% CI	*p*	HR	95% CI	*p*
CD68 (high/low)	4.798	1.883 to 12.23	0.002 *	0.995	0.889 to 1.102	0.934
PU.1 (high/low)	1.906	0.755 to 4.822	0.165	0.995	0.881 to 1.086	0.937
CD163 (high/low)	7.734	3.003 to 19.92	<0.0001 *	1.181	1.019 to 1.435	0.046 *
CD66b (high/low)	1.605	0.632 to 4.073	0.312	1.172	0.993 to 1.377	0.051
CD56 (high/low)	1.103	0.437 to 2.785	0.835	1.029	0.979 to 1.077	0.189
CD20 (high/low)	0.650	0.258 to 1.638	0.367	0.671	0.399 to 0.872	0.024 *
CD3 (high/low)	1.650	0.649 to 4.195	0.271	1.045	0.922 to 1.206	0.501
CD8 (high/low)	4.670	1.836 to 11.88	0.002 *	0.938	0.772 to 1.116	0.453
FoxP3 (high/low)	1.256	0.498 to 3.165	0.6263	0.414	0.055 to 1.980	0.3133
iNOS (high/low)	0.319	0.089 to 1.143	0.2404	0.005	0.00001 to 0.395	0.0402 *

* indicates *p*-values of statistically significant differences.

## Data Availability

Raw sequence data and metadata are available at the NCBI BioProject database under project ID PRJNA838259.

## Supplementary Materials

The following supporting information can be downloaded at: https://www.mdpi.com/article/10.3390/biomedicines10071516/s1, Figure S1: Differential abundance analysis: Heatmap. The selected benchmark is used here to calculate and visualize the significant changes in the microbial communities of renal cell cancer (ccRCC—light cell, papRCC—papillary, chRCC—chromophobic) and normal tissues (norm). Heatmap showing the abundance variation of 170 bacterial taxa at the genus level based on Euclidian distance measure and Ward clustering algorithm. The rows represent the bacterial taxa and columns are the samples. Figure S2: Differential abundance analysis: Volcano plot. The selected benchmark is used here to calculate and visualize the significant changes in the microbial communities of normal tissues and renal cell cancer. Volcano plot showing fold changes of 170 bacterial taxa at the genus level between normal tissue and cancer samples on X-axis and the negative logarithm (base 10) of the p-value on Y-axis. Dashed vertical lines reflect the filtering criteria (Log2 fold change = ±0.5). Green dots represent Genus entities that are significant based on Specific test (LEfSe) at each group. The Grey and Green dots represent the Genus features either common between groups or classified as insignificant by the test (LEfSe). The X-axis represents the abundance fold change on log2 scale, and the Y-axis represents the negative log10 of the calculated *p*-value. NS means non-significant.
