# Supplementary figures and images for: Macrophage Phenotype in Combination with Tumor Microbiome Composition Predicts RCC Patients’ Survival: A Pilot Study
Source: Biomedicines. 2022 Jun 27;10(7):1516. doi: 10.3390/biomedicines10071516 (PMC9312790; doi:10.3390/biomedicines10071516)

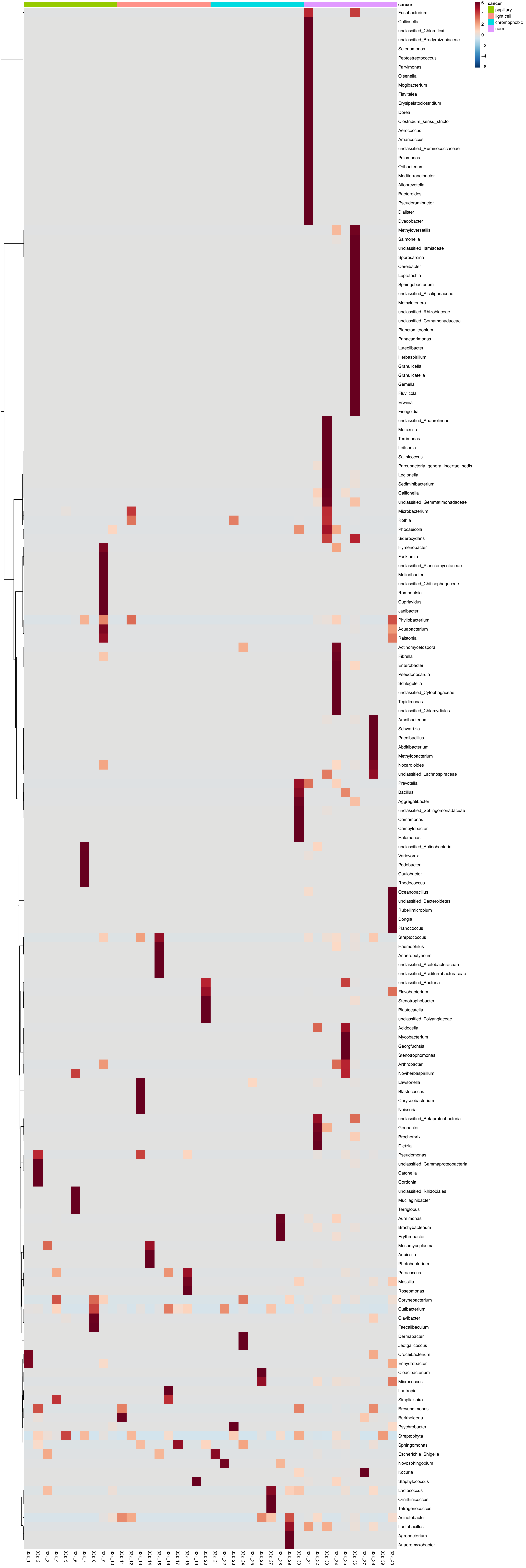

Supplement: Supplementary file 1 [file biomedicines-10-01516-s001.zip › Supplementary figure S1.pdf]

## Volcano plot

EnhancedVolcano

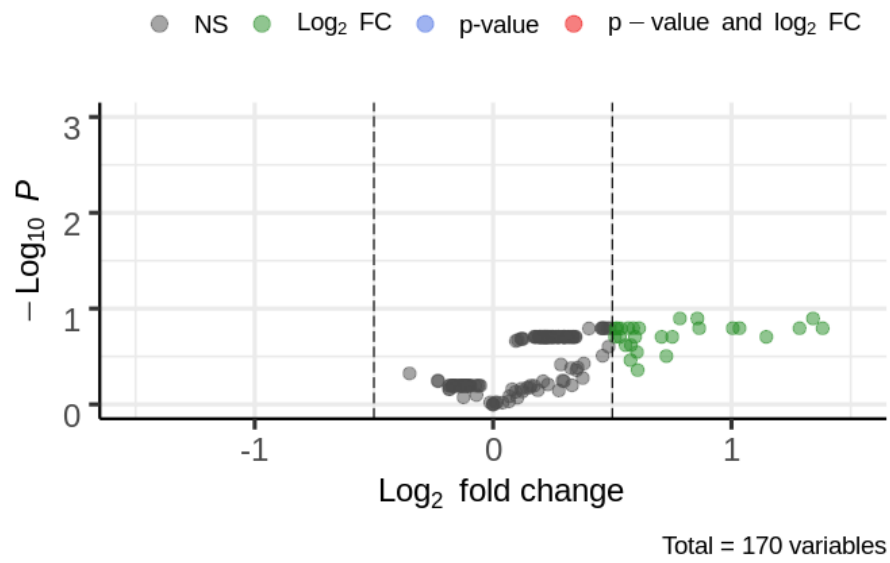

Supplementary figure S2. Volcano plot of 16S sequencing results.

Supplement: Supplementary file 1 [file biomedicines-10-01516-s001.zip › Supplementary figure S2.pdf]
